# Radical Hydrofunctionalization Using Light Driven, Reductive Cobalt Hydride Catalysis

**DOI:** 10.1021/acs.accounts.6c00516

**Published:** 2026-09-30

**Authors:** Hanqi Zhou, He Chen, Christopher J. Teskey

**Affiliations:** 26527 Technische Universität Braunschweig, Institute of Organic Chemistry, Hagenring 30, 38106 Braunschweig, Germany

## Abstract

First row transition metals such as iron, cobalt and manganese with weak-field ligands are able to form metal hydride species in situ which have a weak M–H bond. In these cases, the formal addition of a hydrogen atom to an olefin is thermodynamically favored. The high regio and chemoselectivity of this process, termed metal hydride hydrogen-atom-transfer (MHAT) catalysis, together with the broad availability of olefins and the divergent reaction pathways from the carbon centered radical that is formed, has resulted in a wide range of radical hydrofunctionalization strategies that have been used extensively in complex synthetic settings.

Until around 5 years ago, a typical catalytic cycle for forming the metal hydride relied on oxidation as a first step, followed by reaction with a hydride equivalent. The requirement to find compatible oxidant/reductant pairs defined and limited the scope of this chemistry. However, several groups including our own recognized that it would instead be possible to first *reduce* the metal and then protonate the low-valent form to yield the same metal hydride species. This net reductive approach would unlock new avenues for onward reactivity, circumventing the compatibility issues associated with the reagents used previously.

A number of different approaches to this reductive MHAT paradigm have been reported, using chemical reductants, electrochemistry or photochemistry. Our group has focused on the merger of cobalt catalysis and photocatalysis to enable new hydrofunctionalization reactivity. Under reductive reaction conditions, it is possible to simultaneously form a persistent radical via single electron reduction and couple this with the radical formed from the alkene. We have used this concept to couple alkenes with cyanoarenes, pyridyl phosphonium salts, ketones and imines.

Radical rearrangements, initiated by MHAT, can take place that were previously inefficient or not possible under oxidative MHAT conditions. We have used this to promote aryl migration reactions, building new C–C bonds with sterically demanding aromatic groups. And most recently, we have reported the combination of MHAT catalysis with reductive radical polar crossover, a new type of reactivity that enables the use of an electrophile as a coupling partner, contrasting with the commonly reported oxidative radical polar crossover pathway where hydrofunctionalization takes place with nucleophiles.

In summary, the reductive MHAT concept has reinvigorated interest in radical hydrofunctionalization reactions. Here, we present our journey in uncovering new reactivities accessible only through this catalytic paradigm, enabled by photochemistry. We are confident that both the synthetic utility and the broader appeal of this modern approach to classical MHAT reactivity will continue to increase in the coming years, driven by the growing demand for catalytic systems that employ earth-abundant transition metals and which enable direct functionalization of native olefin functionality.

## Graphical abstract

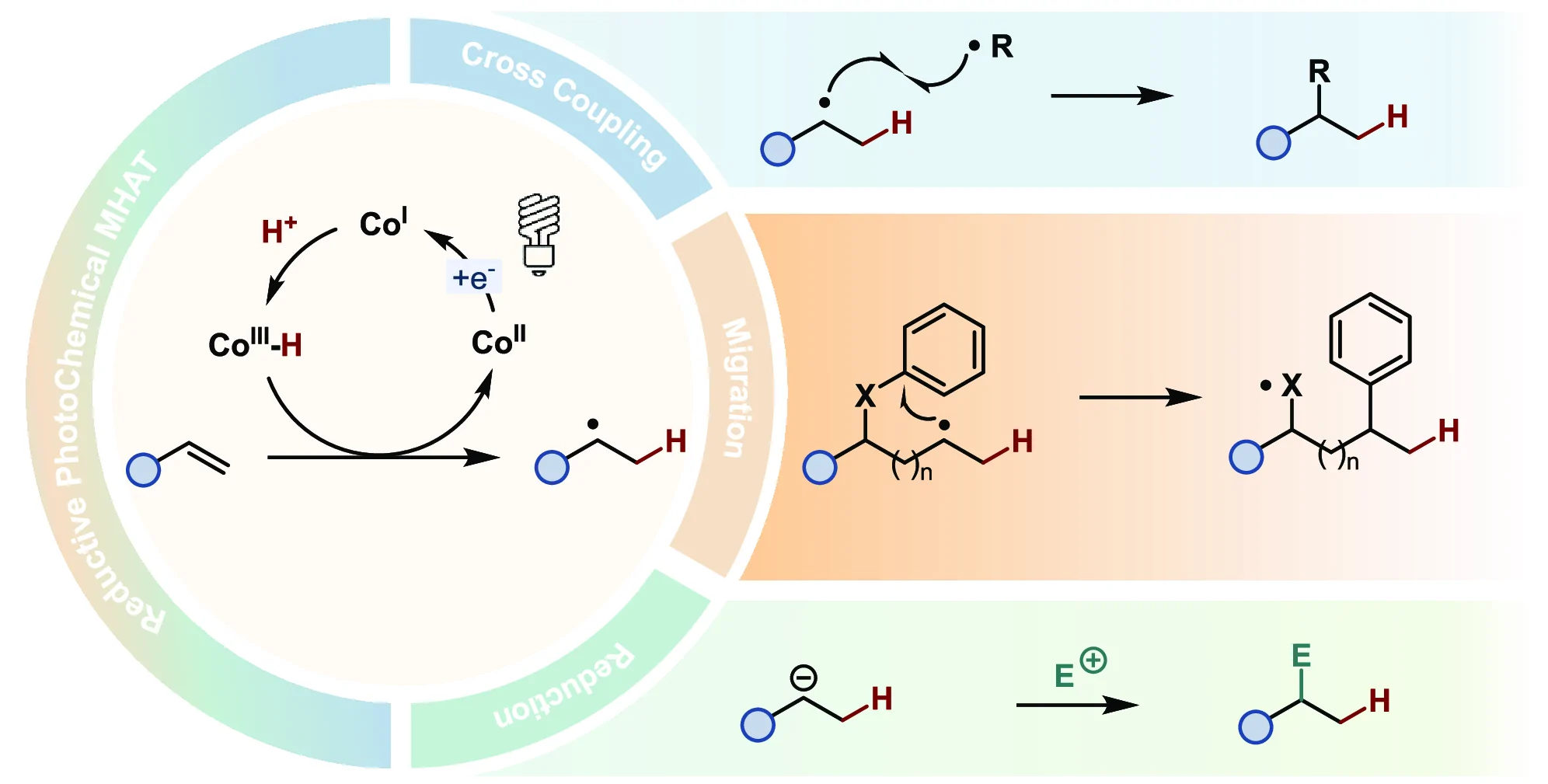

## Key References

-

Bergamaschi, E. ; Mayerhofer, V. J. ; Teskey, C. J. Light-Driven Cobalt Hydride Catalyzed Hydroarylation of Styrenes. ACS Catal.. 2022, 12, 14806–14811 10.1021/acscatal.2c05109 .^1^ Direct excitation of Hantzsch Ester allows it to act successively as an electron and proton donor to a cobalt catalyst. This promotes a sequence of MHAT and subsequent reaction with the radical anion derived from cyanopyridines and cyanoarenes.
-

Qin, J. ; Barday, M. ; Jana, S. ; Sanosa, N. ; Funes-Ardoiz, I. ; Teskey, C. J. Photoinduced cobalt catalysis for the reductive coupling of pyridines and dienes enabled by paired single-electron transfer. Angew. Chem. Int. Ed. 2023, 62, e202310639 10.1002/anie.202310639.37676106 ^2^ Dienes and pyridyl phosphonium salts can be coupled with excellent and predictable regioselectivity. The reaction is initiated via hydrogen atom transfer from a cobalt porphyrin catalyst which can be used in very low loadings.
-

Mayerhofer, V. J. ; Lippolis, M. ; Teskey, C. J. Dual-catalyzed intermolecular reductive coupling of dienes and ketones. Angew. Chem. Int. Ed. 2024, 63, e202314870 10.1002/anie.202314870 37947372.^3^ The coupling of ketones and imines with dienes is enabled through reductive MHAT catalysis. This enables unfavorable O-centered radical intermediates to be bypassed and can be applied to the synthesis of a number of functional molecules.
-

Lunic, D. ; Vystavkin, N. ; Qin, J. ; Teskey, C. J. Dual-catalytic structural isomerization as a route to α-arylated ketones. Angew. Chem. Int. Ed. 2024, 63, 202409388 10.1002/anie.202409388 38977417.^4^ Photoinduced, reductive turnover of a cobalt catalyst, allows the isomerization of allylic alcohols into α-aryl ketones via an initial hydrogen atom transfer step.
-

Jana, S. ; Kusza, D. A. ; Vystavkin, N. ; Lunic, D. ; Teskey, C. J. Inverting polarity in a cobalt MHAT reaction via reductive catalytic turnover. Chem. Sci. 2026, 17, 1098–1104 10.1039/D5SC06119A PMC1262800641268520.^5^ The switch to a reductive MHAT paradigm allows for the merger with reductive radical polar crossover (RRPC). This enables hydrofunctionalization of styrenes with CO_2_ as an electrophilic coupling partner.

## Introduction

Carbon centered radicals are versatile intermediates that can be generated from a broad range of precursors. The developments in photoredox catalysis over the last decades has played a big role in enabling their formation at lower temperatures, under milder conditions and subsequently with broader functional group tolerance.^6^ By developing reactions which operate under conditions which tolerate more heavily functionalized starting materials, it becomes easier to build complexity in fewer steps and form key bonds at later stages of syntheses. As such, radical chemistry has now become an irreplaceable tool for constructing complex organic molecules.^7^

Alkenes are an attractive starting point for radical chemistry as many methods exist for their synthesis. To form a radical from an alkene without addition of specific functional groups or elongation of the carbon skeleton demands addition of a simple hydrogen atom. In analogy to two electron logic Bronsted acid catalysis that forms the more stabilized cation, formal addition of H^•^ forms the more stabilized radical species that can further react. To mediate this challenging hydrogen atom addition to the π-system of an olefin, transition metals have been used as catalysts in what is termed metal-hydride hydrogen atom transfer (MHAT).^8^ In particular, first-row transition metals have proved to be effective and those examples containing weak-field ligands allow this step to proceed in a thermodynamically favorable process with unactivated olefins.^9^

Curiously, the recognition of this mechanistic picture developed only after pioneering synthetic early work in this field. This subsequently leads to a large number of new hydrofunctionalization reactions that have been used at later steps of total synthesis, exemplifying the broad utility of this approach.^10^ One aspect that the majority of methods had in common was that they proceeded through a catalytic cycle that can be broadly simplified as follows: the metal catalyst was oxidized (e.g., Co­(II) to Co­(III)) by an oxidant such as a peroxide or an *N*-fluoro salt. Following ligand exchange with a hydride source, most commonly silanes in state of the art work, the key metal hydride intermediate (e.g., Co­(III)–H) is formed. These conditions had proven exceptionally effective for a range of different reactions, enabling C–H, C–C, and C–heteroatom bond formation through diverse radical-trapping and oxidative radical–polar crossover pathways.^8^ However, the need for a stoichiometric amount of oxidant and compatible oxidant/reductant pair constrained the choice of substrates, coupling partners and catalysts, thereby substantially limiting the reaction diversity. As such, we became interested in the development of orthogonal conditions for the MHAT catalytic cycle that we speculated could unlock a range of new radical hydrofunctionalization reactions.^11^

We took inspiration from dual-catalytic hydrogen evolution systems whereby single electron transfer (SET) from a photocatalyst reduces cobalt catalysts before protonation yields a Co­(III)–H species.^12^ Although this then typically reacts further with a second equivalent of proton to form H_2_ (gas), we hypothesized that with the correct ligand structure, MHAT may instead be favorable. In 2021, Matsunaga and co-workers demonstrated that this was the case in pioneering work.^13^ Reduction of alkene double bonds occurs via a reductive catalytic cycle using ascorbic acid as an electron and proton donor in combination with photocatalysis. Our aim was to develop this paradigm into a platform that would enable unreported reactivity, with a focus on C–C bond formation.

We began with investigating Hantzsch Ester as a (photoactive) reagent which could potentially be both the electron and proton source for reduction and protonation of the cobalt catalyst, hypothesizing that structurally diverse Hantzsch Esters could allow for easy tuning of key properties such as redox potentials and p*K*_a_. In this account, we report developments from our group in this area in the context of the rapidly developing field and show how photocatalytic reductive MHAT catalysis has opened new doors for radical hydrofunctionalization.

### 1 Coupling with Persistent Radicals

Our first idea came from the idea that under reductive conditions it should be possible to also form persistent radicals in the reaction mixture by single electron reduction which would offer a range of alternative coupling partners for radical hydrofunctionalization.^14^ With this in mind, we began with well-known persistent radical precursors: cyanopyridines.^1^ Our hypothesis was that the strongly reducing excited state of Hantzsch Ester, achieved through visible light irradiation, could simultaneously reduce Co­(II) to Co­(I) while also reducing cyanopyridine to its radical anion. The resulting radical cation of the Hantzsch Ester is known to be extremely acidic and so could act as a proton source for Co­(I) to form the key Co­(III)–H intermediate.^15^ MHAT to an alkene would then be followed by selective radical–radical coupling with the persistent radical. After elimination, products of Markovnikov hydropyridylation would be obtained. Notably, existing methods for light-induced hydroarylation reactions proceed in an anti-Markovnikov manner as the first step is aryl radical formation and addition to the π-system.^16^ In contrast, our strategy would reverse this selectivity.

With this in mind, we began by investigating the coupling of styrene with 4-cyanopyridine (Scheme 1a). Although photoredox catalysts, including [Ir­(ppy)_3_] and 4-CzIPN were investigated, the reaction proceeds efficiently in the absence of a photocatalyst, through direct excitation of Hantzsch Ester. The desired product was obtained in good yield using only 1 mol % of the commercially available [Co­(salen^*t*Bu,*t*Bu^)], together with 3 equiv of Hantzsch ester (HEH) and pyridine as the base. These results demonstrated that Hantzsch esters alone can serve a dual role, acting both as the reductant and proton source, hereby directly generating the key Co­(III)–H intermediate without the need for additional photocatalyst.

**Scheme 1:**
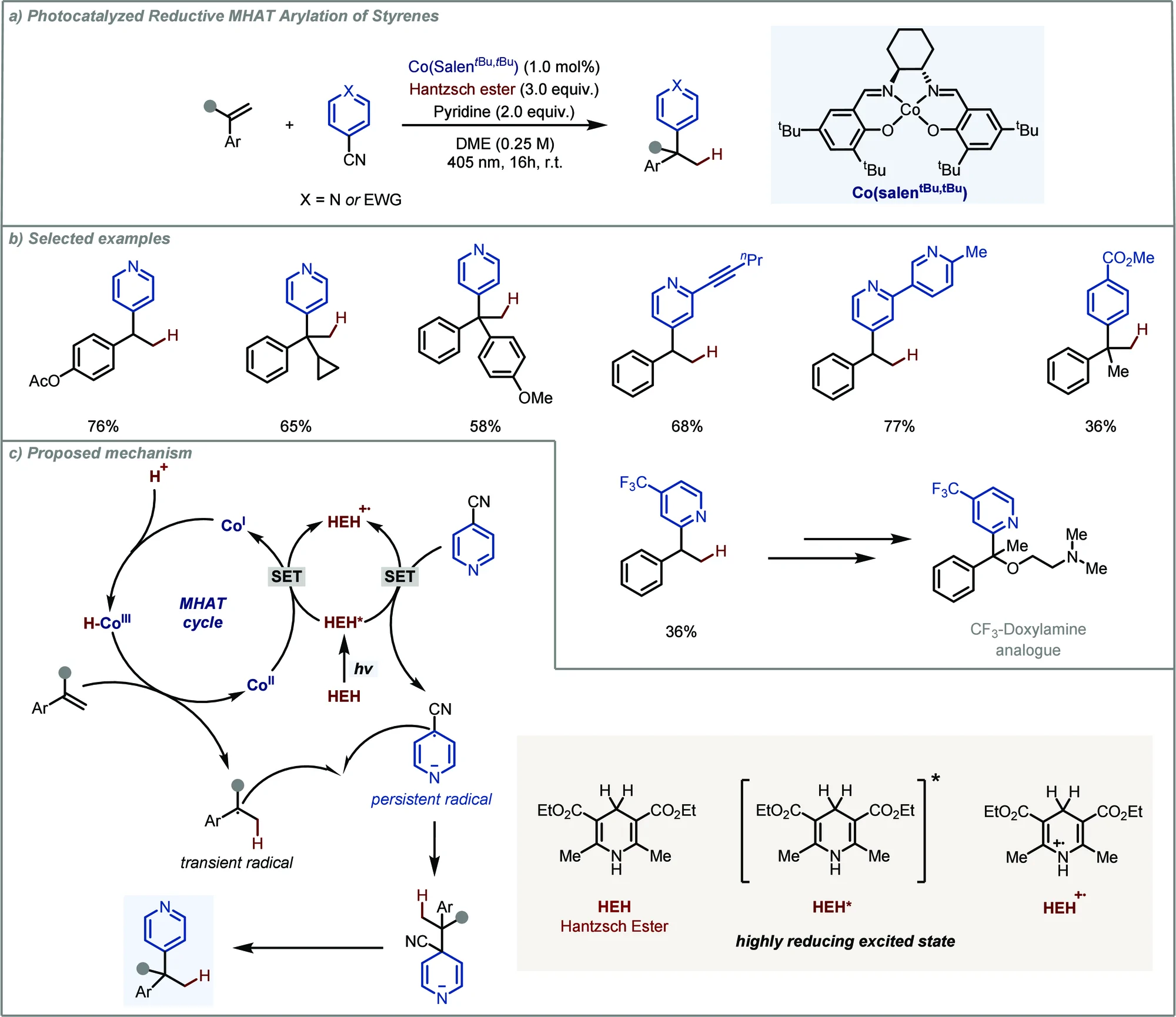
Photocatalyzed Reductive MHAT Arylation of Styrenes

The mechanism takes place as follows (Scheme 1c): the Hantzsch ester is directly excited by visible light (405 nm), generating the strong reductant HEH* (*E*_ox_* = −2.28 V vs SCE in DMF). HEH* can then be quenched by 4-cyanopyridine (*E*_red_ = −1.87 V vs SCE in MeCN) and Co­(II) (*E*_red_ = −1.50 V vs SCE in DMF), forming the persistent pyridyl radical and Co­(I). The Co­(I) species deprotonates HEH^+•^ to generate the key Co­(III)–H intermediate, which adds to styrene to produce a benzylic radical. Finally, the transient benzylic radical selectively couples with the persistent pyridyl radical as a result of the persistent radical effect,^14^ followed by elimination of cyanide, to afford the desired hydroarylation product.

Our method exhibits a broad substrate scope (Scheme 1b), enabling the efficient synthesis of a wide range of 1,1-diarylalkanes and triarylalkanes, including challenging examples bearing three distinct aryl groups. Functional group tolerance is also good, including alkynes, esters and aryl halides. Synthetic utility of this protocol is further exemplified by its application to the preparation of a novel analogue of the antihistamine drug doxylamine. In a concurrent, related study, Lin and co-workers reported a reductive MHAT hydroarylation process using either electrocatalysis or stoichiometric magnesium as the electron source and acetic acid as the proton source.^17^ Similarly to our system, single-electron reduction of cyanoarenes generates persistent aryl radical precursors that couple efficiently with MHAT-generated alkyl radicals. It is worth noting that most examples require 8–10 equiv of magnesium as the reductant, and electrocatalytic conditions often lead to slightly lower yields, however the scope of alkenes is generally wider.

Following this work, we sought to expand our methodology to include a broader range of pyridine molecules as well as diversifying the structure of the alkene coupling partner.^2^ To this end, we investigated if pyridyl phosphonium salts could be productive starting materials under our reaction conditions, based on the precedent from McNally and co-workers that they can also undergo radical–radical coupling with benzylic radicals.^18^ At the same time, we hypothesized that dienes would be interesting starting materials for formation of carbon centered radicals due to their widespread availability (Scheme 2a). However, among these are a number of naturally occurring polyenes which contain diene motifs, as well as alkenes, that are challenging to react selectively under classical MHAT conditions due to unselective HAT (Scheme 2b). An additional point for consideration with dienes is regioselectivity, as subtle differences in diene substitution can strongly bias the site of hydrogen atom transfer. Consequently, precise control over the MHAT process is essential to suppress undesired alkene functionalization. Dienes present one further challenge in terms of selectivity which arises from the intermediate that is formed upon MHAT. A transient allylic radical has two possible sites of reactivity leading to further potential complications and the possibility of undesired side reactions (Scheme 2b). Despite these inherent challenges, we reasoned that the combination of a persistent aryl radical precursor with a carefully tuned MHAT manifold could provide the level of control required for selective diene hydroarylation.

**Scheme 2:**
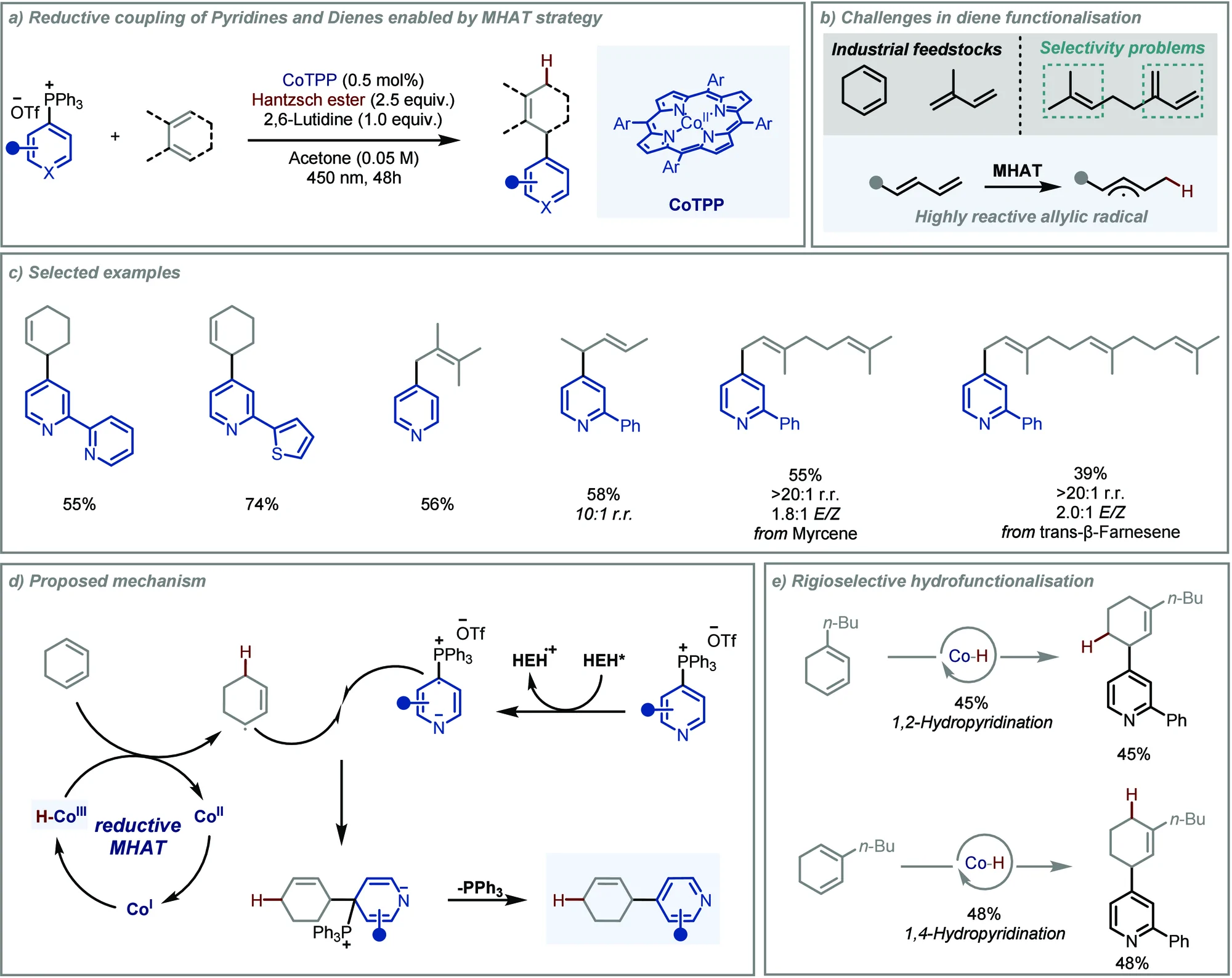
Cobalt-Catalyzed MHAT for Selective Reductive Coupling of Pyridines and Dienes

After extensive investigation, we identified reaction conditions that exhibit exceptional efficiency, requiring only 0.5 of a cobalt porphyrin catalyst under 450 nm light irradiation. Co­(II) is initially reduced to Co­(I) *via* single-electron transfer, followed by protonation to generate the key Co­(III)–H species. This metal hydride undergoes MHAT with 1,3-dienes, regenerating Co­(II) and forming the corresponding allylic radical. The resulting transient radical then selectively couples at the C4 position of pyridine *via* the persistent radical derived from single electron reduction of the pyridyl phosphonium salt. Subsequent extrusion of neutral triphenylphosphine delivers the desired hydroarylation product (Scheme 2d).

Interestingly, the application of the reductive MHAT conditions reported by Lin and co-workers with cyano­(hetero)­arenes did not yield product, resulting only in reduction of the C–P bond of the pyridyl phosphonium starting material.^17^ Oxidative MHAT conditions that had been reported by Herzon and co-workers for hydropyridylation yielded complex mixtures of products.^19^ In contrast, under our optimal conditions, the reaction exhibits exceptional regioselectivity and chemoselectivity. The Co­(III)–H species reacts reliably at the more electron-rich and less hindered terminus of the diene, engaging exclusively with the diene system leaving other simple olefins present in the molecule untouched. For example, complex terpene polyolefins like myrcene and *trans*-farnesene react with complete regioselectivity. Despite the implementation of reductive conditions, reducible functional groups, such as aryl halides and ester groups, are fully tolerated (Scheme 2b).

Introducing substitution on the 1,3-cyclohexadiene moieties allowed us to further probe the selectivity of the hydrofunctionalization (Scheme 2e). Interestingly, the same product was obtained when either 1,*n*-butylcyclohexadiene or 2,*n*-butylcyclohexadiene was employed. These experiments further highlight that hydrogen-atom transfer from the Co­(III)–H species occurs at the sterically less hindered and more electron-rich terminus of the diene. The resulting delocalized allylic radical subsequently couples with the radical anion of the phosphonium salt at the least hindered carbon atom. These observations exemplify the excellent and predictable selectivity of this method.

Building on these two projects, we identified ketones as an alternative category of persistent radical precursors. Coupling would lead to tertiary alcohols, and those containing two aryl groups are key structural motifs in numerous classes of pharmaceuticals.^20^ Accordingly, the development of efficient synthetic methods to access these building blocks continues to attract interest in the community. The intermolecular hydroxylation of 1,3-dienes with carbonyl compounds represents an especially attractive strategy for the construction of tertiary alcohols from readily available starting materials. While related transformations involving aldehydes or aldehyde surrogates have been reported,^21–24^ analogous reactions with ketones remain significantly more challenging, a limitation that likely arises from their diminished electrophilicity and increased steric hindrance. Furthermore, oxidative MHAT relies on the concept of adding a carbon-centered radical to the carbonyl, leading to an oxygen centered radical intermediate which is inherently less stable than the starting radical. As such, it becomes tricky to drive this reaction forward with subsequent limitations to aldehydes or intramolecular reactivity.^25^

Guided by prior studies, we reasoned that our reductive MHAT platform could overcome these challenges (Scheme 3a).^3^ We would develop a catalytic system which again formed allylic or benzylic radicals *via* MHAT from Co­(III)–H, which would be formed *via* a reductive cycle. Importantly, we reasoned that these reductive conditions should permit ketones to undergo single-electron reduction to form the corresponding radical anions. Following HAT to the diene to generate an allylic radical, subsequent radical–radical coupling *via* the persistent radical effect becomes feasible, thereby circumventing unproductive O-centered radical pathways that have limited previous approaches.

**Scheme 3:**
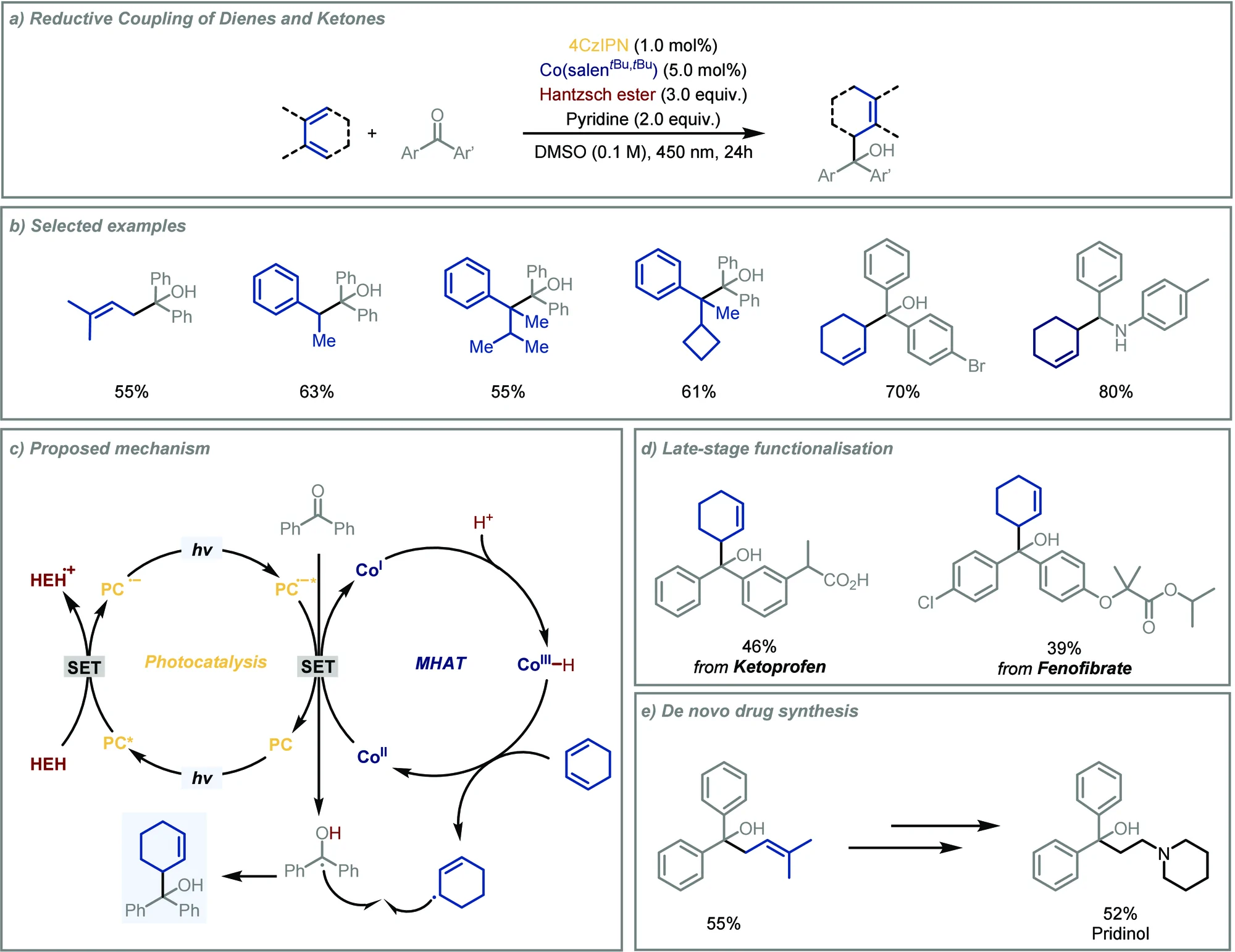
Reductive Coupling of Dienes and Ketones via Cobalt-Catalyzed MHAT

Based on prior literature and our experimental observations, we proposed the following mechanism (Scheme 3c). Upon irradiation at 450 nm, 4CzIPN is photoexcited and undergoes single-electron transfer with the Hantzsch ester (HEH) to generate 4CzIPN^•^ and HEH^•^+^^, as supported by Stern–Volmer quenching studies. Although 4CzIPN^•^–^^ is not sufficiently reducing to undergo SET with benzophenones, alternative pathways such as consecutive photoinduced electron transfer (conPET)^26^ or ligand substitution of the photocatalyst^5^ could lead to more reducing intermediates which are capable of SET. In parallel, 4CzIPN^•^–^^ reduces Co­(II) to Co­(I), which upon protonation forms the key Co­(III)–H species. Subsequent HAT to the diene generates an allylic radical that selectively couples with the ketyl radical anion to deliver the desired tertiary alcohol product.

After extensive optimization, conditions were identified which use 3 equiv of HEH, 5 mol % of the commercially available [Co­(salen^*t*Bu,*t*Bu^)] catalyst, pyridine as the base, and 1 mol % of 4CzIPN as the photocatalyst. The reaction showed broad compatibility with various diene and styrene partners, including α-substituted styrenes, which enabled the construction of congested tertiary alcohols. The observed regioselectivity is consistent with preferential HAT at the more electron-rich and less hindered terminus of the diene. In addition, the reaction could be extended to imines under modified conditions, providing access to amine products (Scheme 3b). Encouraged by the broad functional-group tolerance of this method, we next turned our attention to more complex, functionalized molecules. For example, substrates bearing carboxylic acid and ester groups, such as Ketoprofen and Fenofibrate, underwent reductive coupling with cyclohexadiene to afford the corresponding late-stage functionalization products (Scheme 3d). From a synthetic perspective, we further demonstrated the utility of the products through downstream transformations. In particular, Pridinol could be accessed by oxidative cleavage followed by reductive amination, providing a 3-step synthesis from isoprene and benzophenone that avoids the use of stoichiometric organometallic reagents (Scheme 3e).

### 2 Radical Mediated Aryl Migration Enabled by Reductive MHAT Catalysis

Rearrangement reactions are an important class of transformations that can reorganize molecular skeletons through the cleavage and reformation of chemical bonds. With the renewed interest in radical chemistry over the last decades came correspondingly increased research into radical-mediated rearrangements.^27,28^ Among these, radical aryl migration reactions represent an efficient strategy for relocating an aryl group from a carbon or heteroatom to a radical center through 3-, 4-, 5-, or 6-membered spirocyclic intermediates or transition states (Scheme 4).^29–31^ Notably, a broad diversity of rearrangements have been reported which are initiated by radical addition to an olefin. However, it follows that products are then limited to containing the functional group which begins the process, commonly (fluorinated) alkyl groups. Far fewer examples have been reported which amount to hydrofunctionalization of the double bond where no elongation of the carbon chain or addition of new functional groups is necessary. However, we postulated that MHAT would open up possibilities in this area, with reductive MHAT perhaps allowing for catalyst turnover of reactions that could be carried out only stoichiometrically under oxidative MHAT conditions.

**Scheme 4:**
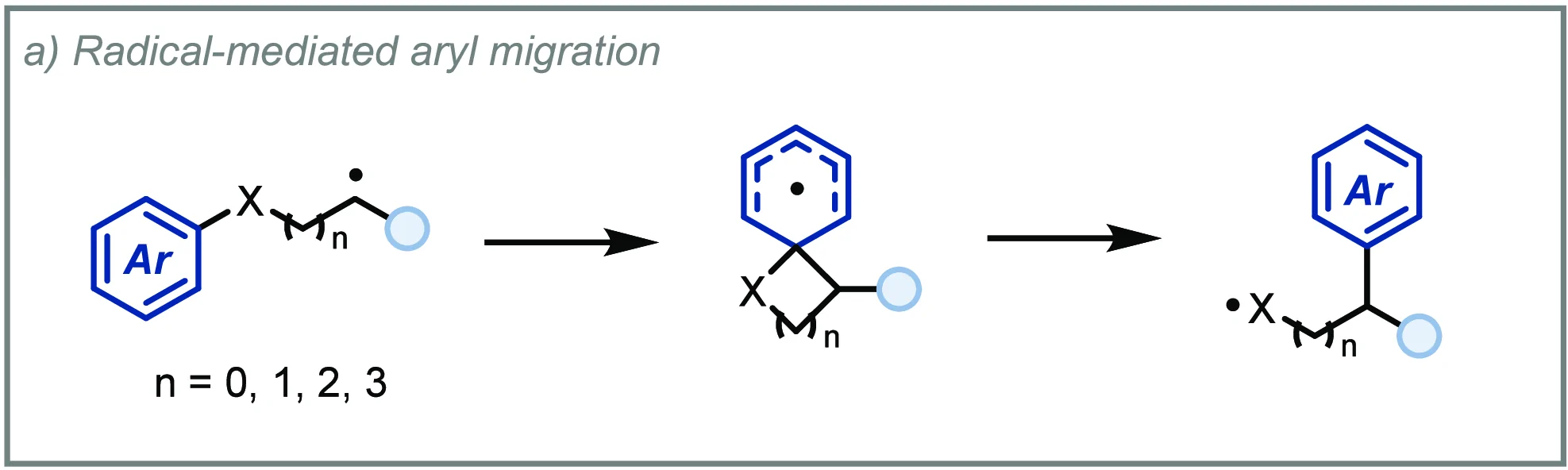
Radical Mediated Aryl Migration

Our attention turned first to 1,2-aryl migration from tertiary allylic alcohols to yield α-arylated ketones. This caught our interest for several reasons: first the products are significantly more challenging to synthesize than the starting materials, with syntheses typically relying on palladium catalysis where regioselective arylation at the more substituted carbon is tricky to control; second, it marked an interesting case to benchmark reductive MHAT against an oxidative variation which had been reported previously but where catalyst turnover was not possible.^32^

Based on the conditions which we had previously developed in our group, we carried out extensive optimization for this MHAT initiated semipinacol type rearrangement (Scheme 5a).^4^ After extensive screening, the cyclohexyl-substituted Hantzsch Ester (HEH-Cy) was found to be optimal, improving yields over the parent compound. We attributed this to the irreversible nature of single electron transfer (SET) step. Additionally, no byproducts are observed from radical addition of the nucleophilic cyclohexyl radical generated from the fragmentation of HEH-Cy to the olefin due to a polarity mismatch. Notably, according to control experiments, AcOH appears to play a key role in proton exchange rather than HFIP which had also been reported as a possible proton source.^33^

**Scheme 5:**
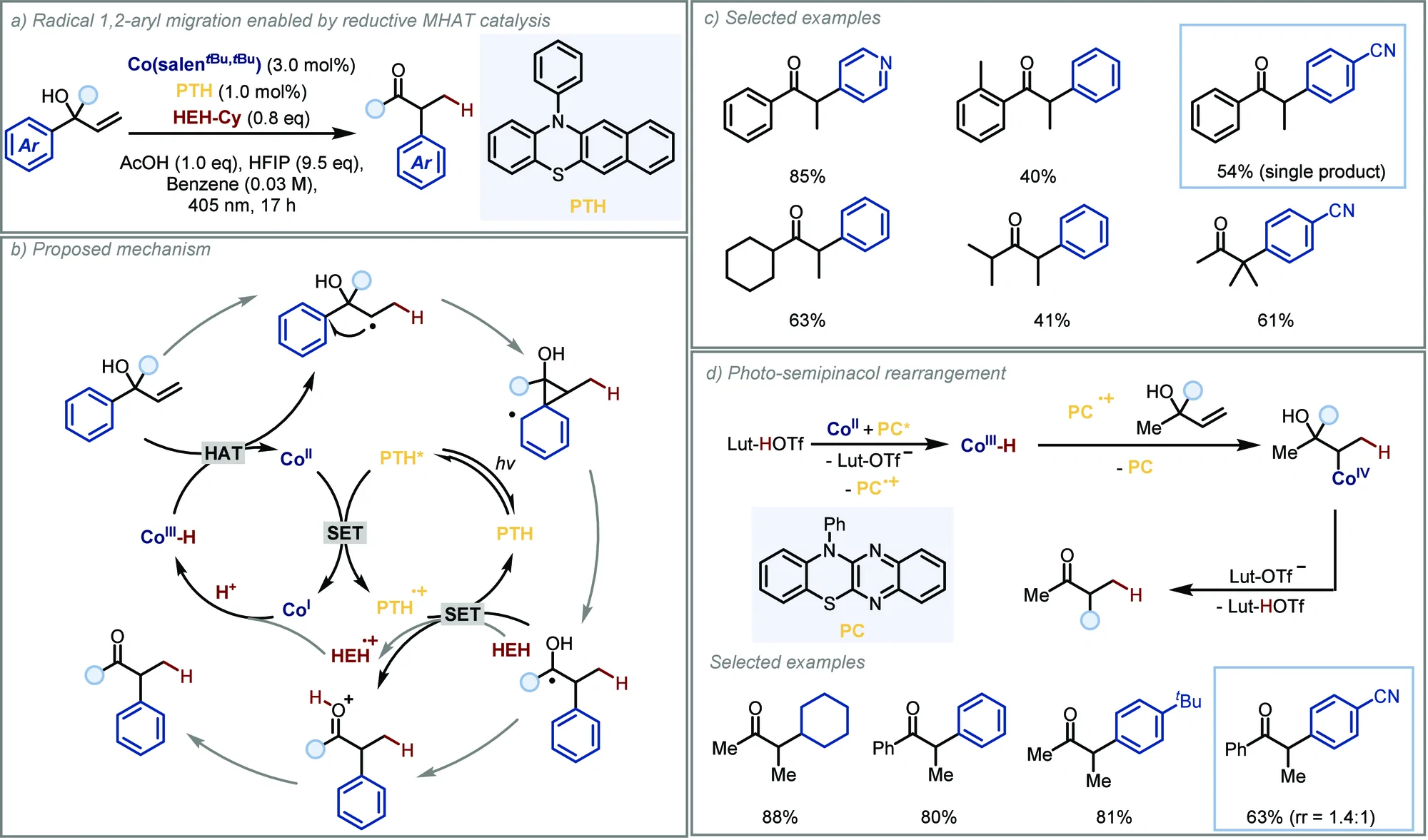
1,2-Aryl Migration of Allylic Alcohols via Reductive MHAT Catalysis

A proposed mechanism is shown in Scheme 5b. Initially, the photocatalyst is excited and reduces Co­(II) to Co­(I). The radical cation of the photocatalyst is subsequently reduced by the Hantzsch ester to return to its ground state. The radical cation of the Hantzsch ester is then deprotonated by Co­(I) to form the key Co­(III)–H species, which undergoes hydrogen atom transfer (HAT) with the allylic alcohol to form the corresponding alkyl radical intermediate. The following rearrangement begins with intramolecular radical attack of alkyl radical on the adjacent aryl group. After ring opening, the resulting protonated ketyl radical is then oxidized by a single electron transfer (SET) pathway to deliver the desired α-arylated ketone after deprotonation.

A radical migration mechanism would explain preferential migration of heteroaromatic and electron-deficient aryl rings over a competing phenyl group in diaryl allylic alcohols, a selectivity that can be rationalized by their lower aromatic stabilization energies.^29^ This is consistent with what we observe in the case of intramolecular competition, except in the case of *ortho*-substituted aromatic rings which do not undergo migration at all. To evaluate the reaction with a less stable ketyl intermediate, substrates bearing a single aromatic ring were investigated. Different alkyl substituents, such as cyclohexyl and isopropyl gave desired products in good yields. Moreover, the product containing a quaternary center at the α-position was also obtained under our conditions (Scheme 5c). Although these initial observations were consistent with a radical rearrangement, the matched-mismatched effects observed with chiral vinyl alcohol substrates and enantiopure cobalt catalysts indicate that the involvement of an analogous cobalt-bound intermediate cannot be excluded.

Simultaneously, Carreira and co-workers reported a similar strategy.^34^ As this transformation amounts to an isomerization, the reaction should also only require a catalytic amount of proton source and electrons to initiate the reaction with the protons and electrons in subsequent cycles being provided by a reaction intermediate. This work demonstrates that this is possible, however, with a suggested difference in mechanism to our work. The authors suggest an oxidative radical-polar-crossover strategy and thus the following mechanistic divergence: a Co­(III)–alkyl intermediate forms after hydrogen atom transfer between Co–H species and allylic alcohol and subsequent cage collapse of the carbon-centered radical and Co­(II). Oxidation by the radical cation of the photocatalyst then forms the key Co­(IV)–alkyl intermediate and a cationic semipinacol rearrangement occurs to deliver the α-arylated ketones. Notably, it can be seen that different selectivities are observed on the same substrate with less clear preference for migration of the more electron deficient arene under these conditions (Scheme 5, highlighted substrate). However, this differing mechanism results in efficient rearrangement also of aliphatic and electron-rich aromatic groups which is not possible under our conditions (Scheme 5d). Notably, this Co­(IV)–alkyl intermediate has also been exploited shortly after by Johnston and co-workers in the enantioselective synthesis of α-aryl carbonyl compounds under oxidative MHAT conditions.^35^

The Truce–Smiles rearrangement is another transformation that we aimed to exploit in the context of our photoinduced MHAT catalysis. It is one of the most prominent synthetic strategies for the translocation of (hetero)­aryl groups from a heteroatom to a carbon atom and has proven to be an effective tool in modern synthetic chemistry for building new C*sp* ^3^-aryl bonds.^36^ Conventional Truce–Smiles rearrangements proceed via an ionic pathway. As the Meisenheimer intermediate contains a negative charge, located on the aromatic ring, substitution by strongly electron-withdrawing groups at the *ortho* and/or *para* positions are necessary.^37^ In contrast, radical variants, the first example of which was reported by Speckamp and co-workers,^38^ have a broader possible scope of migrating aryl groups which do not require additional stabilization. This has directed renewed interest toward this classical rearrangement. In recent years, elegant approaches were reported by Nevado,^39–42^ Stephenson,^43^ Zhu,^44,45^ Greaney,^46^ Masson,^47^ and others to achieve alkene difunctionalization reactions, triggered by initial radical addition and followed by arylation. However, most examples rely on amide- or styrene-derived alkenes, limiting diversity of the carbon framework while initiation often occurs by radical addition to an olefin which necessitates inclusion of specific functional groups. Notably, a single example of MHAT promoted Truce–Smiles rearrangement had previously been reported under oxidative conditions but catalytic turnover was again not possible.^48^

Radical-mediated, Truce–Smiles rearrangements of simple, unactivated *N*-arylsulfonyl allylanilines to access simple, linear β-arylethylamines remained largely unexplored which prompted our investigations (Scheme 6a).^49^ Initial results showed that the radical intermediate can follow three different reaction pathways, leading to desired product via *ipso*-addition to the migrating arene or undesired cyclic side-products by *ortho* addition to either the migrating arene or to the other aromatic substituent on nitrogen (Scheme 6a).^50^ To suppress the undesired *ortho* cyclization, we evaluated the influence of different substituents at the *ortho* position of the migrating groups in detail. DFT calculation revealed that the presence of at least one *ortho* substituent on the migrating group destabilizes the ground state energy of the starting material, bringing it closer to the transition state energy for radical *ipso* attack. In this case, the transition state of *ipso* addition becomes the most stable (13.4 kcal mol^–1^) compared to the competing *ortho* additions (14.8 kcal mol^–1^ and 15.1 kcal mol^–1^) (Scheme 6b). As such, unwanted reaction pathways are effectively circumvented when *ortho*-substituted substrates are employed. The reaction showcased good functional group tolerance and broad substrate scope, delivering the corresponding β-arylethylamines regardless of the electronic character or steric hindrance. Substrates bearing *ortho*-substituted aromatic rings on nitrogen provided slightly lower yields, due to incomplete conversion (Scheme 6c).

**Scheme 6:**
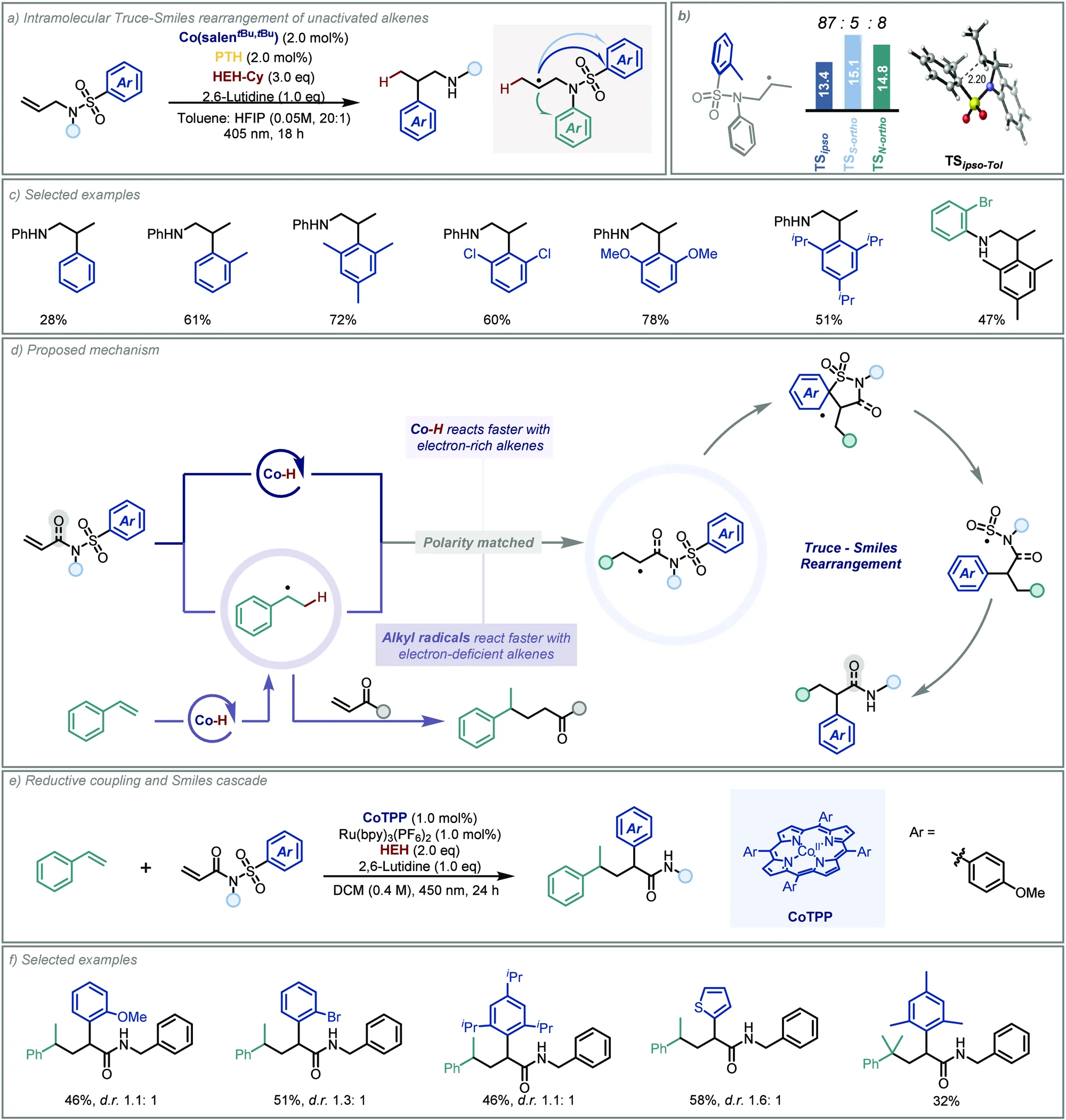
Truce–Smiles Rearrangement of Allylsulfonamides via Reductive MHAT Catalysis

A plausible reaction mechanism is shown in Scheme 6d. After the polarity-matched HAT between electron-rich alkenes (*N*-allylsulfonyl allylanilines) and the Co­(III)–H species, which is generated from the reduction and protonation of a Co­(II)-salen catalyst, an alkyl radical intermediate is formed. The alkyl radical then selectively attacks the *ipso* position of the sulfonyl aromatic ring. From the spirocyclic intermediate, a nitrogen centered radical is subsequently generated from a rapid homolytic fragmentation of C_Ar_-S bond and *N*-desulfonylation, ultimately delivering the desired β-arylethylamines after reduction and protonation.

The observation that MHAT was inefficient to more electron deficient alkenes^9^ fueled the idea that we could potentially merge cross-olefin coupling with the Truce–Smiles rearrangement by polarity matching of reaction intermediates. This would then enable a radical cascade reaction that could form several bonds in a single step.^51^ Cross-olefin coupling *via* MHAT catalysis had been reported previously by Baran and co-workers using iron catalysis.^52^ This proceeds on the basis that the iron hydride species selectively undergoes polarity-matched hydrogen atom transfer (HAT) with electron rich alkenes. The *in situ* generated nucleophilic alkyl radicals subsequently participate in Giese type addition to electron deficient alkenes.

Inspired by this idea and our previous results, we investigated a coupling between styrenes and *N*-sulfonyl acrylamides (Scheme 6e).^53^ The acrylamide derivatives contain a C–C double bond that is significantly more electron deficient than the styrene while also having an arylsulfonyl moiety that could undergo aryl migration. The radical cascade reaction is thus initiated by polarity-matched MHAT between styrenes and the cobalt hydride species generated *via* reduction and protonation of Co­(II). After the formation of the nucleophilic benzylic radical, a Giese type addition occurs to electron deficient alkene. When *N*-sulfonyl acrylamides are used as Michael-acceptors, the generated α-amide radical attacks the *ipso* position of the migrating group, delivering α,γ-diarylated amides *via* Truce–Smiles rearrangement (Scheme 6d). Under the optimized conditions, the cross-olefin coupling/Truce–Smiles rearrangement radical cascade reaction affords a series of α,γ-diarylated amides in moderate yields with a diverse range of migrating (hetero)­aryl groups. Moreover, α-methyl styrene is also compatible with this reaction, yielding the corresponding α,γ-diarylated amide containing a quaternary carbon (Scheme 6f).

Pyridine derivatives are used in most of the reactions which we have developed and likely act not only as a base but also modulate the Co–C bond strength of intermediates.^54^ As such, we carried out mechanistic experiments to probe the effects of pyridine on a cobalt­(III)-benzyl porpyhrin intermediate in this reaction. Here, we observe that pyridine promotes Co–C bond cleavage as well as yielding a notable red-shift of the organocobalt intermediate which additionally promotes photoinduced homolysis at the wavelength used in the reaction. However, it is also important to note that more sterically hindered pyridine derivatives, such as 2,6-lutidine, are often more effective compared to pyridine itself and that there is no concrete evidence that Co–C bonds play a significant role in the reactions we have developed.

### 3 Merger of Radical-Polar-Crossover Chemistry with Reductive MHAT Catalysis

The last branch of reactivity which we have investigated to date is the merger of MHAT catalysis with reductive radical polar crossover. Here, it is clear from the name that a reductive system is required and hence this reactivity would be hard to imagine under classical oxidative MHAT conditions. Radical polar crossover is, however, well-known and precedented in the oxidative form. The Co­(III)–alkyl species is often reported to undergo single electron oxidation to the corresponding Co­(IV)–alkyl species whereby the carbon is rendered electrophilic in character, with reactivity similar to a free carbocation.^55^ As such, reaction with nucleophiles can occur, directly mirroring Brønsted acid catalysis but with an additional element of catalyst control which has led to some powerful enantioselective reactions.^56,57^

Interestingly, in recent years, pioneering studies by Shigehisa,^58,59^ Pronin,^60,61^ Ohmiya,^62–64^ Lin,^65^ Carreira^34,66^ and others have significantly expanded the versatility of oxidative radical-polar-crossover reactions, some even combining the reductive MHAT catalytic cycle such that cobalt proceeds through oxidation states (I) to (IV) (Scheme 7a). In contrast, no report existed that merged reductive radical-polar-crossover reactions with MHAT catalysis, a concept that would enable hydrofunctionalization with electrophiles by inverting the polarity of the internal carbon through single electron reduction of the alkyl radical intermediate (Scheme 7a). With the development of reductive MHAT catalysis, we hypothesized that we now had a catalytic platform that would be capable of mediating this type of reactivity.

**Scheme 7:**
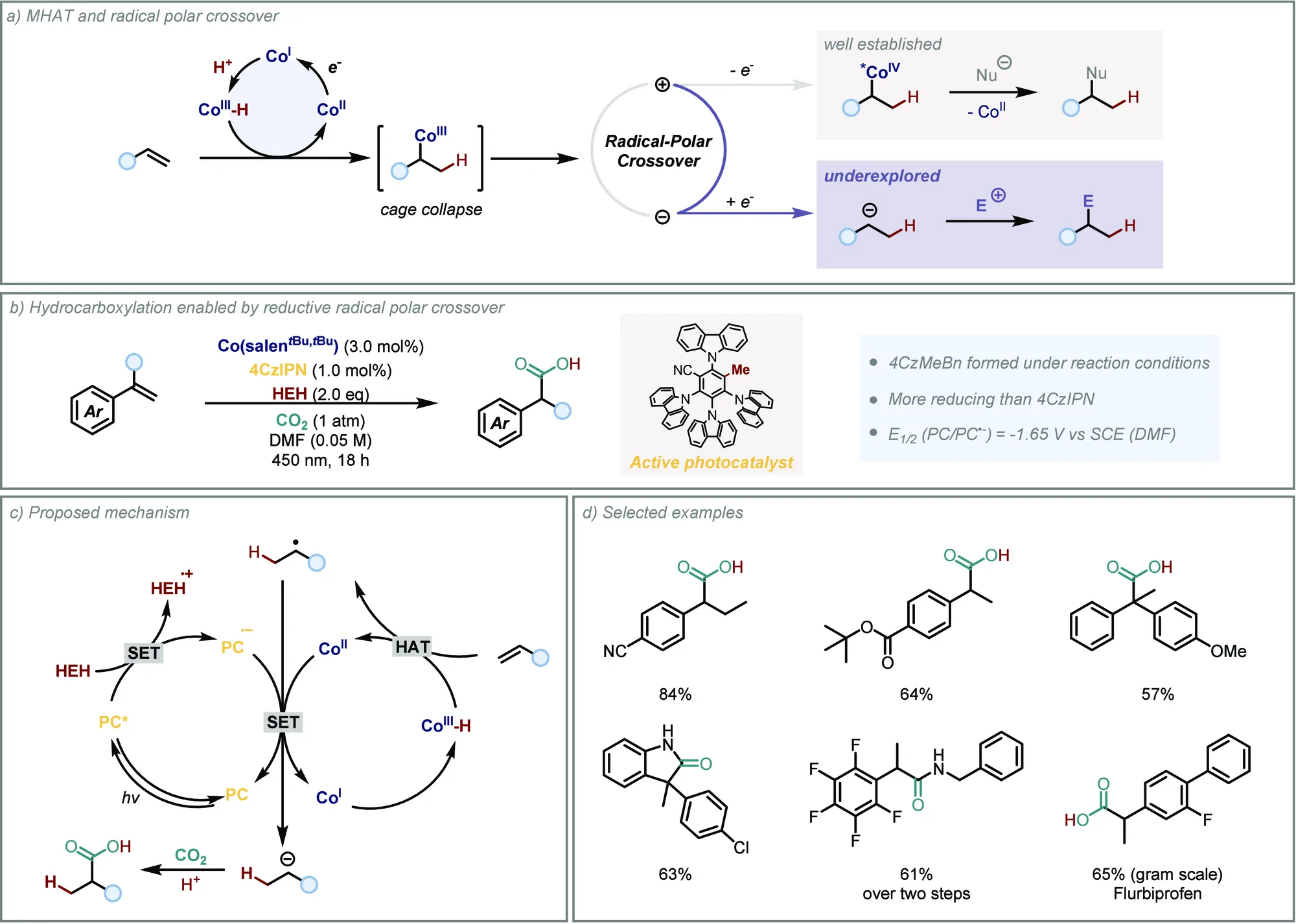
Radical-Polar-Crossover Reactions with MHAT Catalysis

Fine tuning of the photocatalytic, reductive cobalt hydride catalysis developed by our group allowed us to develop a new catalytic platform for Markovnikov-selective hydrocarboxylation using carbon dioxide as the electrophile (Scheme 7b).^5^ The developed reaction proceeds as follows: the photocatalyst 4CzIPN (or 4CzMeBN, a stronger reducing species that is formed in situ *via* methyl substitution of a nitrile group) is excited and then quenched by Hantzsch ester to generate the radical anion of the photocatalyst. This undergoes single electron transfer with the metal catalyst to form Co­(I). Subsequent protonation yields Co­(III)–H, which then undergoes MHAT with styrene to yield a benzylic radical intermediate. After single electron reduction by the radical anion of the photocatalyst, the key anionic species is generated and subsequently reacts with carbon dioxide to deliver the Markovnikov branched carboxylic acids (Scheme 7c). Under the optimized conditions, the reactions proceeded smoothly with electron deficient and neutral styrenes derivatives. Unfortunately, no desired products were obtained with the electron rich styrenes, likely due to the more challenging reduction of the benzylic radical intermediate. Additionally, we successfully extended the scope to 1,1-diaryl olefins, which afforded the hydrocarboxylation products in high yields. Interestingly, *ortho*-NH_2_-substituted substrates give γ-lactams via *in situ* cyclization in good yields. Finally, we were able to showcase the applicability of our method to the gram-scale synthesis of pharmaceutically relevant compounds, Flurbiprofen, a nonsteroidal anti-inflammatory drug (Scheme 7d).

Shortly after our report, a similar reaction catalyzed by cobalt and a photocatalyst was reported by Gryko and co-workers.^67^ Their approach instead uses sodium formate as the C1 building block in combination with *N*,*N*-diprotonated DABCO salt which can function as both a Brønsted acid and hydrogen-atom-transfer reagent to enable the Markonikov selective hydrocarboxylation of a diverse range of styrene and diene derivatives.

## Conclusion

In the last 5 years, our group has developed a range of new synthetic methods which combine photocatalysis and cobalt catalysis to promote MHAT under orthogonal conditions to those used earlier. Our concept centers around the use of Hantzsch Esters which are yellow solids that can either be directly irradiated or function in combination with a photocatalyst. These reagents play a dual role as both a source of protons and electrons to promote reductive turnover of the cobalt catalyst. This overcomes the limitations of oxidant/reductant compatibility that previously restricted applications of MHAT catalysis. This reaction platform could be generalized to a range of different transformations, significantly broadening the diversity of radical hydrofunctionalization. It has enabled integration with persistent radicals as coupling partners, that are formed via single electron reduction, as well as new radical rearrangements and reductive radical polar crossover to allow hydrofunctionalization with an electrophile. Our work has focused on the formation of new C–C bonds and combining simple starting materials to form more complex organic molecules.

Despite rapid advances in recent years, several challenges in the field remain to be addressed. Current methods are generally less effective for polysubstituted and internal alkenes, where steric congestion slows MHAT and often results in more complex regio- and stereochemical outcomes which restricts utility. Side reactions such as hydrogenation and isomerization may interfere with further development of metal-hydride-mediated processes and homocoupling of radical intermediates is sometimes observed. We envisage that future advances will evolve from the development of new ligand classes and use of alternative transition-metal catalysts beyond cobalt that enable more precise control and expand the scope of subsequent bond formation. The integration of MHAT with photoredox catalysis, electrochemistry and other catalytic platforms will likely broaden the range of accessible transformations while further reducing reliance on stoichiometric oxidants and silane reagents. Milder conditions should enable hydrofunctionalization in more complex settings, ultimately allowing MHAT catalysis to be used for the modification of biomolecules.
